# Supplementary material for: Patterns of Intron Gain and Loss in Fungi
Source: PLoS Biol. 2004 Nov 30;2(12):e422. doi: 10.1371/journal.pbio.0020422 (PMC532390; doi:10.1371/journal.pbio.0020422)
Supplement: Table S1 — Also available at http://genes.mit.edu/NielsenEtAl/. (4.3 MB ZIP). [file pbio.0020422.st001.zip › NielsenEtAl/html/374.html]

AN6369.1.NCU00290.1.MG08918.1.FG05589.1


```
 CLUSTAL W (1.82) Multiple Sequence Alignments - Introns Inserted


Sequence 1: NCU00290.1	659 aa
Sequence 2: FG05589.1	625 aa
Sequence 3: MG08918.1	629 aa
Sequence 4: AN6369.1	559 aa
Alignment Length: 671 aa
Number Identitical Residues: 270 aa
Alignment Score (without introns) 13688


MG08918.1 	MSAHHGYSEPARQSDHRSEFHSQHASDLGKDGADVESSSASGAHLLNTTVKSISWTNITV
NCU00290.1	------MGISSSSSEHSTSVE---RKDPVVEGIDIERHAVAESHLVNTTVKNISWKGVTV
FG05589.1 	--------MALKGSDHS---------------MDSEQRPVAEKHLVNTTIKNFTWSNVTV
AN6369.1  	--------MACSSSSSSSD---------GLNAPDVEQNAGSHDFLMNHSVQSFSWSDLTV
          	         .   *.  :.           .. * *  . :  .*:* :::.::*..:**

MG08918.1 	KVTDRDTKQPRLILDNVEGIVNAG1ELVALMGPSGCGKTTLLNMLASRPTGAGS-----T
NCU00290.1	TVKDRETKEPKNIVDNVDGIVEAG1EILACMGPSGSGKTTLLNFLASRPLPSASGGGTTT
FG05589.1 	TVKDRETKQPKAIVDDVQGIVEAG~EICALMGPSGCGKTTLLNVLARRPTNASN-----A
AN6369.1  	SVRDRRTKQPLNLIESVSGSVQQG1ELVALMGPSGCGKTTLLNVLARRAASSGAK----S
          	.* ** **:*  :::.*.* *: * *: * *****.*******.** *.  :.      :

MG08918.1 	DGVVLVNGTEPSRAAFRQMSCFVEQEDALIGSLNVRETLMFASRLSTSS~GSLSSKERTR
NCU00290.1	SGSVLINGQPTSTSTFREITRFVEQEDTLIGSLTVRETLDFSFRLASSS~KVLPKRERIA
FG05589.1 	EAQVLANGKHLSLAEFREVSCFVEQEDALIGSLTVRETLEFSSRLASSS2-SLSKKERNV
AN6369.1  	SGHCYVNGGEVDNATFGRITSYVEQEDALIGSLTVRETLKFAADLSLPG2-FVSRSERVE
          	..    **   . : * .:: :*****:*****.***** *:  *: ..   :.  **  

MG08918.1 	RVDGLLAAFGLSDQASTLIGTPIRKGISGGQKRRVGVASQLITSPKILFLDEPTSGLDSA
NCU00290.1	RIEGLLDAFGLRGQKDALIGTPIRKGISGGQKRRVSVASQLITSPRILFLDEPTSGLDSK
FG05589.1 	RIDNLLESFGLVEQANTLIGTPIRKGISGGQKRRVGVASQLITSPKLLFLDEPTSGLDSA
AN6369.1  	RIHTLLESFGIQNQASTLVGTPIRKGISGGQKRRVSVASQLITCPKILFLDEPTSGLDST
          	*:. ** :**:  * .:*:****************.*******.*::************ 

MG08918.1 	ASREVMS~YLKSVAKRNN0--------------------------GKQHYFGPVTEVVPY
NCU00290.1	AGWEVVK~YLKEVARRNN0LIVIASIHQPSTATFNLFDKLMLLSAGKMHYFGPVTGVTEH
FG05589.1 	ASLEVVK~YLRAVAKRNN~LIVICSIHQPSTSTFNLFDKLLLLSGGKTHYFGPVSSVTSY
AN6369.1  	ASYEVIS2----------~--------------------------GKTCYYGPVKQVPSY
          	*. **:.                                      **  *:***. *  :

MG08918.1 	YESIGLDVPVHSNPAEWLLELINVDFAEDRANAQTLLGRLQEAWTSSSRCQQLRAGLRQV
NCU00290.1	YEALGHEVPLHVNPAEFLLDLVNIDFASEREEAVKALDDMQTAWQGSERSRQLTAAVAEA
FG05589.1 	YAQVGAPLPQYVNPAEHLLELVNIDFAQNRGEASRNLGSLQDSWATSQQASQVSDAIKTA
AN6369.1  	FGSIGYPIPPQTNPAEFILDIVSSDFMSEKNSMRNDVEAIQSAWSQSPESSRLKQQRTEF
          	:  :*  :*   **** :*:::. ** .:: .    :  :* :*  * .. ::       

MG08918.1 	ESFGSTGAETLVGSGGHSERKPGQLSLVLTLLHRSFVKSHRDVVAYGIRMAMYTG~LAIM
NCU00290.1	E---ARGGDQVDISTAGLGGKPGLIGSTATLLHRSFVKSYRDVVAYGIRAAMYFG~LAIM
FG05589.1 	E---SSGGD---WSVETIEKRPSMPSLTLTLLHRSFIKSYRDVVAYGIRLAMYLG~LAIM
AN6369.1  	A---EKGSRRVVVDEAS---RPGLLSITISLLHRLLIKSYRDVVAYGIRIVMYMG1LAIM
          	      *.     .      :*.  . . :**** ::**:********* .** * ****

MG08918.1 	MGTVWLRLEPEQSSIQPFINAI~F1F~GSAFMSFMAVAYVPSFIEDRLQYVKEHHNGLYG
NCU00290.1	VGTVWVRLDAAQESIIPFINAL~F~F2GSAFMSFMAVAYCPAWLEDYFQYVKERRNGLYG
FG05589.1 	MGTVWLRLKPEQESIQPFINAI~F~F~GSAFMSFMAVAYVPAFIEDRLQYVKEHHNGLYG
AN6369.1  	MGTVWLRLGTEQEYIQPFINAI0F~F~GSAFMSFMAVAYVPAYLEDRATFTKERANGLYG
          	:****:** . *. * *****: * * ************ *:::**   :.**: *****

MG08918.1 	ATALMISNFLIGIPYLF1LIAISFSAISYWLNNFQPTAHAFFVWVMWVFLDLLAAESLVV
NCU00290.1	PTALIISNFLIGIPYLF~GFSLLFSVISYWLVNFQPTATAFFIWVFWLFCDLLAAESLVV
FG05589.1 	ATELILSNFLIGIPYLF~IISALFSVISYWLSNFQPTATAFFTWVMWLFLDLLAAESLVV
AN6369.1  	ALPFIISNFIIGLPFLF1LISILFSIVSYWLSNFRPSAPAFFTWVMWLFLDLVAAESLVV
          	.  :::***:**:*:**  ::  ** :**** **:*:* *** **:*:* **:*******

MG08918.1 	LFTSLFPSFVISLALVAFANGLWMSVNGFMV1QPTILNVFYKYVFHYWDYQ~KYVFEGMM
NCU00290.1	FMSSLFPSFVISLALVAFANGLWMSVDGFMV~QPTILNVFYKYVFHYWDYQ~KYVFENMM
FG05589.1 	FMTSLFPSFVISLALVAFANGLWMSVGGFMV~PPTILNVFYKYVFHYWDYQ~KYVFEGMM
AN6369.1  	LVTSIFPNFVIALALVAFANGLWMSVGGFLV~SPNILNPFWKYVFHYIDYQ0AYVFQGMM
          	:.:*:**.***:**************.**:*  *.*** *:****** ***  ***:.**

MG08918.1 	INEFADRSYGCG-----------DSCQCM~YVTELADQCKIAGEGVLRQYGYSTGHTGHD
NCU00290.1	INEFKDRVYTCGKLPEGMTGVNGSNCQCM~YQTDLADQCLIRGQGVLDQYGYKPGKQGRD
FG05589.1 	VNEFSKRVYSCG---------NG--CRCM~YQSALADQCKIDGQAVLDQYGYSSGHLGRN
AN6369.1  	VNEFAERIYSCAS--------NGSECNCM2------------------QMGWDPAGDYRR
          	:*** .* * *..        ....*.**                   * *:...   : 

MG08918.1 	VGIMVAIIAGYRIAGWIALKLRT
NCU00290.1	IGIMFCIIVGWRLAAWVVLKLKK
FG05589.1 	VGIMISIIAGYRIAAWLVLVLRR
AN6369.1  	L----------------------
          	:
```
